# Dynamics and Mechanisms of ERK Activation after Different Protocols that Induce Long-Term Synaptic Facilitation in *Aplysia*

**DOI:** 10.1093/oons/kvac014

**Published:** 2022-10-18

**Authors:** Yili Zhang, Rong-Yu Liu, Paul Smolen, Leonard J Cleary, John H Byrne

**Affiliations:** Department of Neurobiology and Anatomy, W.M. Keck Center for the Neurobiology of Learning and Memory, McGovern Medical School at the University of Texas Health Science Center at Houston, 6431 Fannin Street, Suite MSB 7.046, Houston, TX 77030, United States; Department of Neurobiology and Anatomy, W.M. Keck Center for the Neurobiology of Learning and Memory, McGovern Medical School at the University of Texas Health Science Center at Houston, 6431 Fannin Street, Suite MSB 7.046, Houston, TX 77030, United States; Department of Neurobiology and Anatomy, W.M. Keck Center for the Neurobiology of Learning and Memory, McGovern Medical School at the University of Texas Health Science Center at Houston, 6431 Fannin Street, Suite MSB 7.046, Houston, TX 77030, United States; Department of Neurobiology and Anatomy, W.M. Keck Center for the Neurobiology of Learning and Memory, McGovern Medical School at the University of Texas Health Science Center at Houston, 6431 Fannin Street, Suite MSB 7.046, Houston, TX 77030, United States; Department of Neurobiology and Anatomy, W.M. Keck Center for the Neurobiology of Learning and Memory, McGovern Medical School at the University of Texas Health Science Center at Houston, 6431 Fannin Street, Suite MSB 7.046, Houston, TX 77030, United States

**Keywords:** computationally designed training protocol, TrkB, TGF-β, *Aplysia*, long-term facilitation, ERK

## Abstract

Phosphorylation of the MAPK family member extracellular signal–regulated kinase (ERK) is required to induce long-term synaptic plasticity, but little is known about its persistence. We examined ERK activation by three protocols that induce long-term synaptic facilitation (LTF) of the *Aplysia* sensorimotor synapse – the standard protocol (five 5-min pulses of 5-HT with interstimulus intervals (ISIs) of 20 min), the enhanced protocol (five pulses with irregular ISIs, which induces greater and longer-lasting LTF) and the two-pulse protocol (two pulses with ISI 45 min). Immunofluorescence revealed complex ERK activation. The standard and two-pulse protocols immediately increased active, phosphorylated ERK (pERK), which decayed within 5 h. A second wave of increased pERK was detected 18 h post-treatment for all protocols. This late phase was blocked by inhibitors of protein kinase A, TrkB and TGF-β. These results suggest that complex interactions among kinase pathways and growth factors contribute to the late increase of pERK. ERK activity returned to basal 24 h after the standard or two-pulse protocols, but remained elevated 24 h for the enhanced protocol. This 24-h elevation was also dependent on PKA and TGF-β, and partly on TrkB. These results begin to characterize long-lasting ERK activation, plausibly maintained by positive feedback involving growth factors and PKA, that appears essential to maintain LTF and LTM. Because many processes involved in LTF and late LTP are conserved among *Aplysia* and mammals, these findings highlight the importance of examining the dynamics of kinase cascades involved in vertebrate long-term memory.

## INTRODUCTION

Extensive research has delineated ways in which protein kinase A (PKA) and MAPK pathways contribute to synaptic plasticity that is essential for the formation of long-term memory (LTM) [1–15]. Our previous studies have combined computational and empirical approaches to investigate the ways in which PKA and MAPK cascades contribute to the induction phase of LTF at *Aplysia* sensorimotor synapses [16–18]. These studies together with empirical findings from our laboratory and others illustrate a high degree of complexity in the dynamics of these kinase pathways [19–23]. The regulation of transcription factors by kinases [24–29] includes biphasic regulation of kinases and multiple feedback loops [30–34]. Critical roles are played by growth factors [19, 35–44] such as neurotrophin (NT) and transforming growth factor β (TGF-β), which contribute to the ERK activation for at least 1 h after 5-HT treatment [18, 19, 38, 43].

Most empirical studies have focused on the dynamics of ERK activation within a few hours after 5-HT. Little is known about long-term activation of ERK and how it is regulated. The present study examined these issues using three protocols that induce LTF. These protocols have substantial differences in the timing and duration of 5-HT treatment. The standard protocol (five pulses of 5-HT with regular ISIs of 20 min) is commonly used to induce LTF [16, 21]. The enhanced protocol (five pulses of 5-HT with computationally designed irregular ISIs of 10–10–5–30 min) induced greater and longer-lasting LTF [16] The two-pulse protocol (ISI of 45 min) is derived from a protocol that produces LTM [38, 45, 46].

Here, we used immunofluorescence analysis to characterize the dynamics and mechanisms of activation of ERK up to 24 h after 5-HT application. Based on the rather complex biochemical circuitry that includes cross talk and multiple intracellular and extracellular feedback loops (Fig. 5), we expected to observe complex dynamics in the level of ERK that has been activated by obligatory phosphorylation, which we denote pERK. Moreover, we expected that the dynamic analysis might provide insights into the relative effectiveness of the enhanced protocol in producing LTF. We found a complex dynamic activation of ERK for all three protocols, with a late phase of activation at 18 h. For the enhanced protocol, elevated pERK levels persisted to 24 h. At 18 and 24 h, the late phase of activation was blocked by inhibitors of PKA and TGF-β. An inhibitor of TrkB also blocked the late phase at 18 h and partially blocked it at 24 h. These results suggest that complex interactions among kinase pathways and growth factor cascades contribute to the late increase of pERK, which is likely important for maintaining late LTF. In general, the results emphasize the importance of elucidating the dynamics of kinase cascades for understanding neuronal and synaptic plasticity.

## MATERIALS AND METHODS

### Empirical methods

#### Neuronal cultures

No vertebrate animals were used in the research. All experiments used primary cultures of identified sensory neurons (SNs) from *Aplysia californica* (NIH *Aplysia* resource facility, University of Miami, Miami, FL). *Aplysia* are hermaphrodites. Animals were maintained in circulating artificial seawater at 15°C. SNs were isolated from the ventral–caudal cluster of the pleural ganglion from 60 to 100 gm *Aplysia* according to conventional procedures [17, 18, 28]. Each dish of SN cultures was plated with 5–10 SNs. SNs were allowed to grow for 5–6 days at 18°C before experiments begun, and the growth medium was replaced at least 2 h prior to treatments with a solution of 50% L15 and 50% artificial seawater (ASW; 450-mM NaCl, 10-mM KCl, 11-mM CaCl_2_, 29-mM MgCl_2_, 10-mM HEPES at pH 7.6).

#### Immunofluorescence analysis

Immunofluorescence procedures for SNs followed those of Zhang *et al.* [17, 18]. Briefly, after 5-HT treatment, cells were fixed at specific time points in a solution of 4% paraformaldehyde in PBS containing 20% sucrose. Fixed cells were washed by PBS, and then blocked for 30 min at room temperature in a solution of Superblock buffer (Pierce) mixed with 0.2% Triton X-100 and 3% normal goat serum. Cells were subsequently incubated overnight at 4°C with primary anti-phosphorylated ERK antibody (anti-pERK, Cell Signaling, cat. no. 4370, RRID: AB_2315112, 1: 400). After primary antibody incubation and PBS wash, secondary antibody (goat anti-rabbit secondary antibody conjugated to Rhodamine Red, Jackson ImmunoResearch Lab, cat. no. 111-295-144, RRID: AB_2338028, 1:200) was applied for 1 h at room temperature. Cells were then mounted using Mowiol 4-88 (SigmaAldrich). The images of cells were obtained with a Zeiss LSM800 confocal microscope using a 63× oil-immersion lens. A z-series of optical sections through the cell body (0.5-μm increments) was taken. The section through the middle of the nucleus was used for quantification of mean fluorescence intensity of the whole cell, with ImageJ-win64 software (NIH). All the cells on each coverslip were analyzed and averaged. The number of samples (*n*) reported in Results indicates numbers of dishes assessed.

#### Experimental design

For each of the three protocols, a single pulse of 5-HT consisted of a 5-min application of 50-μM 5-HT (Sigma) to SNs. At the end of the 5-min pulse, the chamber bath was washed with 10 ml of L15/ASW. Dishes of SNs cultured from the same animals were paired for all the 5-HT treatments. One dish received a solution consisting of 50% isotonic L15 and 50% artificial seawater (L15-ASW) as vehicle control (Veh). The other received the same solution with the addition of 5-HT. The experimenter was blind to the identity of treatments given to each pair of dishes. Falcon petri dishes of 50 mm × 9 mm were used.

In the experiments to measure the time course of pERK after 5-HT protocols, each paired dish was either fixed for immunofluorescence immediately after 5-HT, or incubated in L15/ASW after wash off of 5-HT until fixation at specified times (1, 2, 5, 18 and 24 h for standard protocol; 15 min, 1 h, 1.5 h, 3 h, 18 h and 24 h for two-pulse protocol; and 18 and 24 h for enhanced protocol) Fig. 2. The remaining dish served as a time-matched Veh control. For each pair of dishes measured at the same time point, the averaged level of pERK from the dish receiving 5-HT was compared to the averaged pERK from the Veh control.

Application of all the inhibitors began 1 h prior to the fixation for immunofluorescence, to ensure that inhibitors were given sufficient time to penetrate the cells and block the activities of kinases. To examine the effects of PKA activity on pERK, 10-μM cAMP inhibitor Rp-cAMP (Calbiochem) was applied to SN cultures 1 h prior to the fixation (fixed at 18 h for standard and two-pulse protocols; 24 h for enhanced protocol). Previously, 10-μM cAMP inhibitor Rp-cAMP inhibited the increase of pERK ~45 min post-onset of 5-min treatment with 5-HT without affecting basal activity [18]. Four dishes of SNs from the same animals were used for each experiment. Each dish was given a different treatment: (i) 50-μM 5-HT alone, (ii) 10-μM Rp-cAMP alone, (iii) 5-HT + Rp-cAMP or (iv) Veh alone.

To examine the effects of TrkB on pERK,10 μg/ml of a TrkB antagonist, TrkB Fc chimera (acts via receptor sequestration) (TrkB Fc) (R&D Systems), was applied to SN cultures 1 h prior to fixation (fixed at 18 h for standard and two-pulse protocols; 24 h for enhanced protocol). TrkB Fc specifically binds to and neutralizes TrkB ligands, preventing ligand-mediated signaling. Previously, at this concentration, TrkB Fc inhibited the increase of pERK at 1 h after the two-pulse protocol in isolated *Aplysia* SNs without affecting basal activity [18]. Four dishes of SNs from the same animals were used for each experiment. Each dish was given a different treatment: (i) 5-HT alone, (ii) TrkB Fc alone, (iii) 5-HT + TrkB Fc or (iv) Veh alone.

To examine the effects of TGF-β on pERK, 5 μg/ml of TGF-β RII Fc chimera (TGF-β RII Fc) (R&D Systems), was applied to SN cultures 1 h prior to fixation to inhibit TGF-β signaling. At this concentration, TGF-β RII Fc inhibited the increase of pERK at 1 h after two pulses of 5-HT [38]. Four dishes of SNs from the same animals were used for each experiment. Each dish was given a different treatment: (i) 5-HT alone, (ii) TGF-β RII Fc alone, (iii) 5-HT + TGF-β RII Fc, or (iv) Veh alone. The number of samples (*n*) reported in Results indicates the number of animals.

#### Electrophysiology

Excitatory postsynaptic potentials (EPSPs) were recorded from motor neurons (MNs) from the SN–MN co-cultures following established procedures [16, 26–28, 47, 48]. Briefly, the EPSP was evoked in the MN by stimulating the SNs with a brief depolarizing stimulus using an extracellular electrode filled with L15: ASW. Intracellular recordings from MNs were made with 10–20 MΩ sharp electrodes filled with 3 M potassium acetate connected to an Axoclamp 2-B amplifier (Molecular Devices). Data acquisition and analyses of resting potential, input resistance and EPSP amplitude were performed with pCLAMP 8 software (Molecular Devices). Before measurement of EPSPs, MNs were held at −90 mV by passing constant current. Cultures were excluded from further use if pre-treatment measurements of EPSP amplitudes were less than 10 mV, larger than 35 mV, or sufficiently large to trigger an action potential. MNs with resting potentials more positive than −40 mV or input resistances less than 10 MΩ were also excluded. These measurements were repeated at 24 h after treatment. The number of samples (*n*) reported in Results indicates the number of co-cultures.

### Statistical analyses

**Figure 1:**
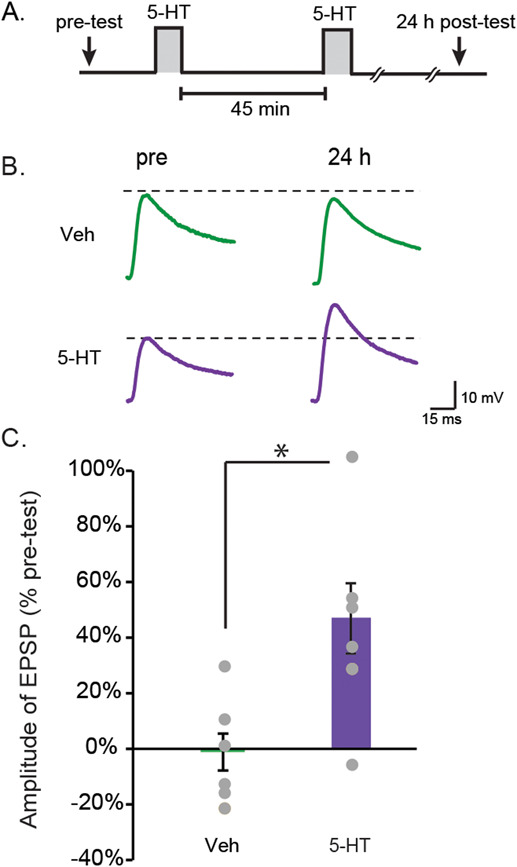
Induction of LTF by the two-pulse protocol. (**A**) Protocol for two-pulse treatment with 5-HT (50 μM). (**B**) Representative EPSPs recorded from MNs in sensorimotor co-cultures before (pre) and 24 h after treatment. (**C)** Summary data. Two-pulse treatment with 5-HT induced significant increases in the amplitude of EPSPs. Bar height represents the mean, small bars represent standard error of the mean (SEM), and significant differences are indicated by ^*^ for *P* < 0.05. Gray circles indicate the results of individual experiments.

**Figure 2:**
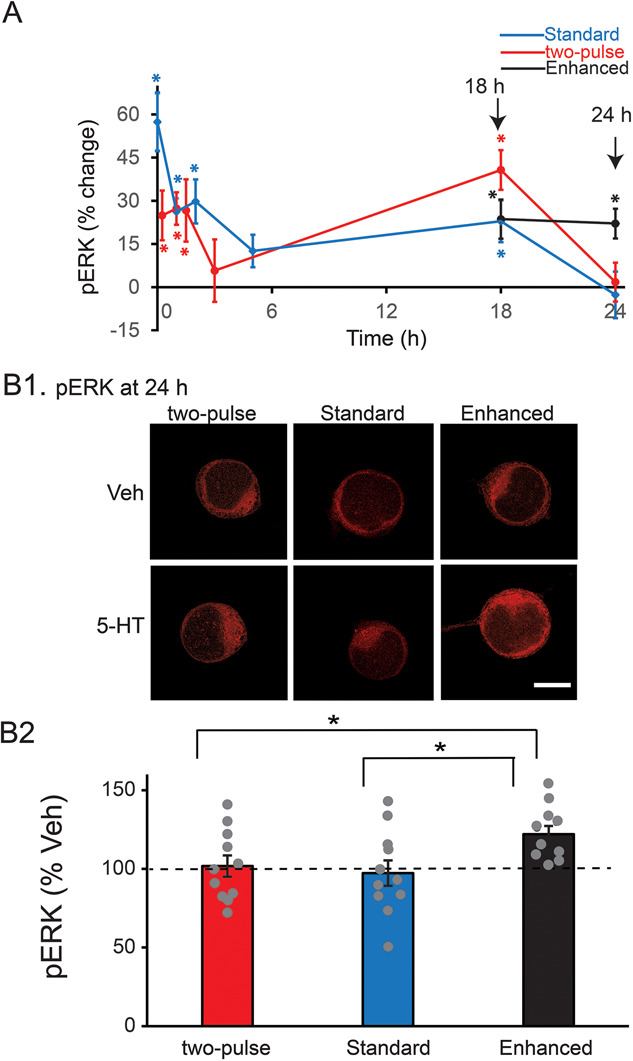
Dynamics of pERK induced by three LTF-inducing protocols. (**A**) Time course of ERK activation. The percent change was calculated as the change of pERK level after 5-HT compared to time-matched vehicle control. A late increase of pERK at ~18 h was induced by the two-pulse protocol (red), standard protocol (blue) and enhanced protocol (black), with no significant differences between protocols at this time point. At 24 h, however, pERK only remained elevated for the enhanced protocol. (**B**) Changes of pERK at 24 h after different 5-HT protocols. (**B1**) Representative confocal images of pERK immunostaining in SNs. (**B2**) Summary data. pERK induced by the enhanced protocol was significantly greater than pERK induced by the other two protocols. Scale bar in **B1** is 40 μm. Bar heights in **B2** represent the mean, small bars represent standard error of the mean (SEM). Gray circles indicate the results of individual experiments. ^*^ *P* < 0.05.

At least five animals were used in each experiment. SigmaPlot version 11 (Systat Software) was used for statistical analyses. Before applying statistical tests, Shapiro–Wilk Normality and Equal Variance tests were performed. In the experiments to compare EPSPs between Veh and 5-HT treatment groups, Student *t*-tests were used (Fig. 1). In the experiments to compare pERK between paired Veh and 5-HT treatment groups at different times, a paired *t*-test with Bonferroni corrections was used for comparison between paired groups if data passed normality and equal variance tests at all time points. Otherwise, a Wilcoxon Signed Rank Test (WSRT) with Bonferroni corrections was used. In Fig. 2A, because data for the normality variance test failed at one time point, the WSRT with Bonferroni corrections was used for comparison of pERK immunoreactivity between paired Veh and 5-HT treatment groups at all time points. Adjusted *P* values after Bonferroni corrections were used to represent statistical significance.

In the experiments to make multiple comparisons of pERK at 24 h between groups treated with three different 5-HT protocols, one-way ANOVA and the *post hoc* Student–Newman–Keuls (SNK) method were used on raw data (Fig. 2B).

**Figure 3:**
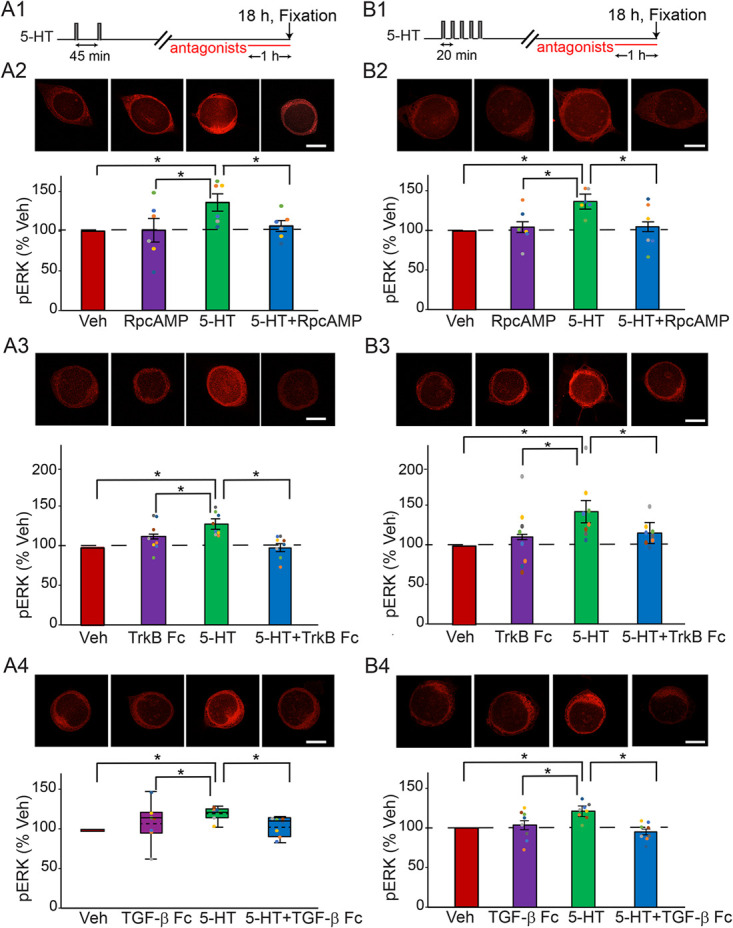
Late increase of pERK at 18 h after the two-pulse (**A**), or the standard protocol (**B**) was dependent on PKA, TrkB and TGF-β. (**A1**) Protocol for applying the antagonists after two-pulse protocol. (**A2**) Representative confocal images and summary data of pERK in SNs at 18 h after two pulses of 5-HT, in the absence or presence of PKA inhibitor RpcAMP. RpcAMP significantly decreased pERK induced by 5-HT (*n* = 6). (**A3**) Representative confocal images and summary data of pERK at 18 h after two pulses of 5-HT, in the absence or presence of TrkB inhibitor TrkB Fc. TrkB Fc significantly decreased pERK induced by 5-HT (*n* = 8). (**A4**) Representative confocal images and summary data of pERK at 18 h after two pulses of 5-HT, in the absence or presence of TGF-β inhibitor TGF-β RII Fc (TGF-β Fc). TGF-β RII Fc significantly decreased pERK induced by 5-HT (*n* = 7). (**B1**) Protocol for applying the antagonists after standard protocol. (**B2**) Representative confocal images and summary data of pERK in SNs at 18 h after five pulses of 5-HT, in the absence or presence of PKA inhibitor RpcAMP. RpcAMP significantly decreased pERK induced by 5-HT (*n* = 6). (**B3**) Representative confocal images and summary data of pERK at 18 h after five pulses of 5-HT, in the absence or presence of TrkB inhibitor TrkB Fc. TrkB Fc significantly decreased pERK induced by 5-HT (*n* = 8). (**B4**) Representative confocal images and summary data of pERK at 18 h after five pulses of 5-HT, in the absence or presence of TGF-β inhibitor TGF-β RII Fc. TGF-β RII Fc significantly decreased pERK induced by 5-HT (*n* = 8). All scale bars are 40 μm. Bar heights in **A2–3**, **B2–B4** represent the mean, small bars represent standard error of the mean (SEM). Circles indicate the results of individual experiments. Circles with the same color indicate the results are from the same animal. Data in **A4** are not normally distributed, thus presented by box-and-whisker plots. The median is indicated by the solid line in the interior of the box. The mean is indicated by the dashed line in the interior of the box. The lower end of the box is the first quartile (Q1). The upper end of the box is the third quartile (Q3). In this and the next figure, the ends of the vertical lines (whiskers) are the maximum and minimum values of all data points. ^*^ *P* < 0.05

**Figure 4:**
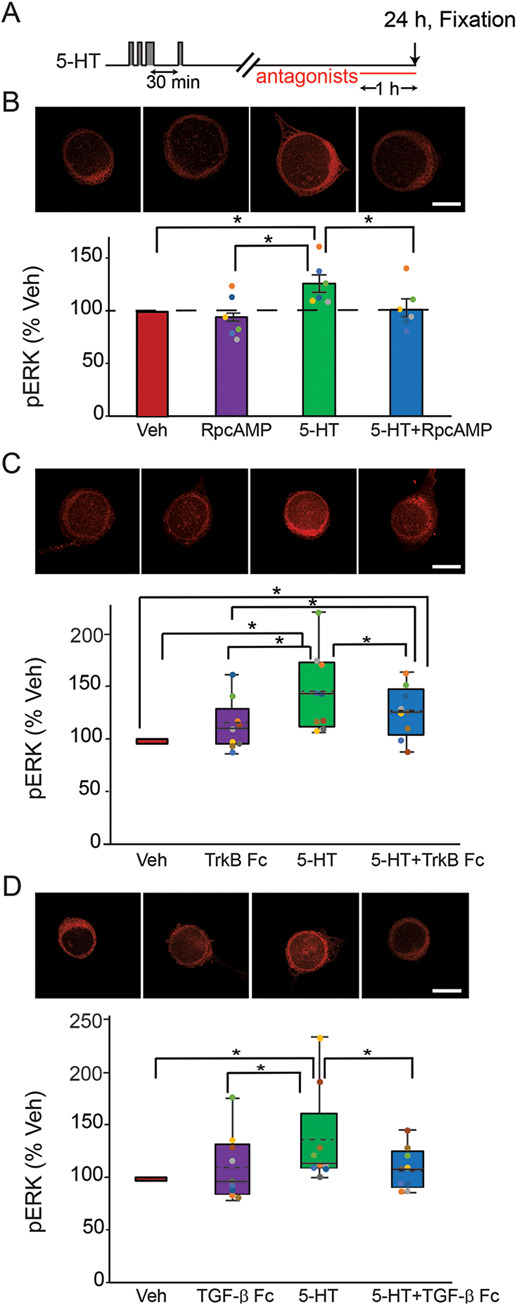
Late increase of pERK at 24 h after the enhanced protocol was dependent on PKA and TGF-β, and partially dependent on TrkB. (**A**) Protocol for applying the antagonists after enhanced protocol. (**B**) Representative confocal images and summary data of pERK in SNs at 24 h after five pulses of 5-HT, in the absence or presence of PKA inhibitor RpcAMP. RpcAMP significantly decreased pERK induced by 5-HT (*n* = 6). (**C**) Representative confocal images and summary data of pERK at 24 h after five pulses of 5-HT, in the absence or presence of TrkB inhibitor TrkB Fc. TrkB Fc significantly decreased pERK induced by 5-HT (*n* = 9). (**D**) Representative confocal images and summary data of pERK at 24 h after five pulses of 5-HT, in the absence or presence of TGF-β inhibitor TGF-β RII Fc. TGF-β RII Fc significantly decreased pERK induced by 5-HT (*n* = 9). All scale bars are 40 μm. Bar heights in **B** represent the mean, small bars represent standard error of the mean (SEM). Circles with the same color indicate the results of individual experiments from the same animal. Data in **C** and **D** are not normally distributed, thus presented by box-and-whisker plots. The median is indicated by the solid line in the interior of the box. The mean is indicated by the dashed line in the interior of the box. The lower end of the box is the first quartile (Q1). The upper end of the box is the third quartile (Q3). ^*^ *P* < 0.05

In the experiments to make multiple comparisons between groups treated with 5-HT and inhibitors, repeated measures one-way (RM) ANOVA and the *post hoc* Student–Newman–Keuls (SNK) method were used on raw data (Figs 3 and 4), except for data displaying a non-normal distribution (Figs 3A3 and 4B and C), which used Friedman repeated measures analysis of variance on ranks and the *post hoc* SNK method.

All of the electrophysiological and imaging experiments were conducted in a blind manner so that the investigator analyzing the data was unaware of the treatment the cells received. A *P* value less than 0.05 was considered to represent statistical significance. The data underlying this article will be shared on reasonable request to the corresponding author.

## RESULTS

### Two-pulse 5-HT protocol induces LTF

LTM can be induced *in vivo* with only two spaced trials if the second tail shock is administered 45 min, but not 15 or 60 min, after the first [45]. The first trial recruits nuclear MAPK activity that establishes a unique molecular context involving the recruitment of p90 ribosomal S6 kinase, a CREB1 kinase downstream of MAPK, and also recruitment of CCAAT/enhancer binding protein (C/EBP), which is required for the induction of LTM [46]. A second trial at 45 min is thought to enhance PKA activity [46] while MAPK activity is still elevated, with the consequent overlap of increased PKA and MAPK activities necessary for LTF/LTM [16]. One likely role of PKA is to activate a heterodimer of C/EBP and *Aplysia* activating factor (ApAF) that is important for consolidation of LTF [49]. In line with this second-trial role, the two-pulse LTM-inducing protocol was also able to produce LTF of the sensorimotor synapse (Fig. 1). EPSP amplitudes were measured before (pretest) and 24 h after (posttest) two, 5-min pulses of 5-HT with an ISI of 45 min (Fig. 1A). Two groups of preparations were examined, a two-pulse group and a vehicle control (control) group, which received two 5-min applications of solution without 5-HT (45-min ISI). Sample recordings are illustrated in Fig. 1B, and summary data are illustrated in Fig. 1C. The two-pulse treatment significantly enhanced the EPSPs (Veh, −2 ± 7%, *n* = 7; two-pulse, 47 ± 13%, *n* = 7; *t*_12_ = 22.06, *P* = 0.006).

### Two-pulse protocol and standard protocol induce two waves of increased pERK within 24 h after 5-HT treatment

Exposure of SNs to different protocols of 5-HT treatment leads to activation of ERK [9, 17, 18, 38, 46], but there is a lack of comparison of detailed dynamics of long-term activation of ERK by different protocols and how ERK activity is regulated by growth factors and kinases that interact with MAPK pathways. Therefore, we used immunofluorescence to examine the dynamics of phosphorylation of ERK induced by two LTF-inducing protocols. We measured levels of pERK 15 min, 1 h, 1.5 h, 3 h, 18 h, and 24 h after two pulses of 5-HT with ISI of 45 min. The early time points within 3 h were selected based on previous studies of the two-pulse protocol [38, 43, 50]. The late time points of 18 h and 24 h were selected because late kinase activation might contribute to differences between the different protocols.

The two-pulse protocol induced two waves of increase in pERK (Fig. 2A, red line). ERK phosphorylation increased 15 min (24.9 ± 8.6%, *n* = 15), 1 h (27.3 ± 5.6%, *n* = 8), and 1.5 h (26.6 ± 10.8%, *n* = 11) after treatment. pERK returned toward basal level at 3 h (5.7 ± 10.9%, *n* = 10). Surprisingly, a late increase in pERK was detected at 18 h (40.7 ± 6.9%, *n* = 8), and then it returned to basal level at 24 h (1.8 ± 6.8%, *n* = 11). Statistical analyses (WSRT, Bonferroni correction) revealed that the increases at 15 min, 1 h, 1.5 h, and 18 h after 5-HT were significant compared to time-matched Veh controls (15 min, *z* = 2.613, *P* = 0.042; 1 h, *z* = 2.521, *P* = 0.048; 1.5 h, *z* = 2.667, *P* = 0.03; 18 h, *z* = 2.521, *P* = 0.048), whereas the changes in pERK levels at 3 h and 24 h were both not (3 h, *z* = 0.153, *P* = 5.532 after Bonferroni corrections; 24 h, *z* = 0.0889, *P* = 5.796 after Bonferroni corrections).

Interestingly, the standard protocol of five pulses of 5-HT with regular ISIs of 20 min induced a similar pattern of dynamic changes in pERK (Fig. 2A, blue line). ERK phosphorylation increased immediately (57.4 ± 9.9%, *n* = 10), 1 h (26.3 ± 4.6%, *n* = 9) and 2 h (29.8 ± 7.6%, *n* = 17) after treatment. pERK returned toward basal level at 5 h (12.6 ± 5.6%, *n* = 10), followed by a late increase at 18 h (22.9 ± 7.3%, *n* = 8) and then a return to basal level at 24 h (−2.7 ± 8.1%, *n* = 11). Statistical analyses (WSRT, Bonferroni correction) revealed that the increases immediately, 1, 2 and 18 h after 5-HT were significant compared to Veh controls (0 min, *z* = 2.803, *P* = 0.012; 1 h, *z* = 2.666, *P* = 0.024; 2 h, *z* = 2.627, *P* = 0.042; 18 h, *z* = 2.521, *P* = 0.048), whereas those at 5 h (*z* = 1.682, *P* = 0.63) and 24 h (*z* = −0.415, *P* = 4.404 after Bonferroni corrections) were not.

### Enhanced protocol leads to elevation of pERK at 24 h post-training

We hypothesized that the enhanced protocol might produce stronger and more persistent LTF if it led to greater activation of pERK at later times. We focused on the 18- and 24-h time points, because preliminary results indicated that at the early time points (0 to 5 h post treatment) the enhanced and standard protocols elicited similar dynamic changes. As with the standard and two-pulse protocols, pERK was elevated at 18 h (23.6 ± 6.8%, *n* = 7) (Fig. 2A, black line). However, in contrast to both the two-pulse and standard protocols, pERK remained elevated at 24 h (22.1 ± 5.2%, *n* = 11). Statistical analyses (WSRT, Bonferroni correction) revealed that the increases at 18 and 24 h after 5-HT were both significant compared to Veh controls (at 18 h, *z* = 2.366, *P* = 0.032; at 24 h, *z* = 2.934, *P* = 0.002). At 18 h there were no significant differences in pERK levels among the standard, enhanced and two-pulse protocols.

To further confirm the differences in ERK activation at 24 h among three protocols, we directly compared pERK levels at 24 h following the three protocols (Fig. 2B), using the same data in Fig. 2A. A one-way ANOVA revealed a significant overall effect of the treatments (F_2,30_ = 3.804, *P* = 0.034). Subsequent pairwise comparisons (Student–Newman–Keuls) revealed that the enhanced group was significantly different from the two-pulse group (*q* = 3.003, *P* = 0.042) and from the standard group (*q* = 3.658, *P* = 0.038). No significant difference was detected between the two-pulse group and the standard group (*q* = 0.655, *P* = 0.647).

### PKA, TrkB and TGF-β pathways mediate the late increase of pERK after the two-pulse and standard 5-HT protocols

Previous studies suggest that the sustained increase of pERK 1 h after the two-trial protocol is, at least partially, dependent on PKA, TrkB and TGF-β pathways [18, 38]. We investigated here whether the late increase of pERK 18 h after 5-HT protocols is also dependent on these pathways.

Three inhibitors were used for these experiments: the PKA inhibitor RpcAMP, the TrkB inhibitor TrkB Fc and the TGF-β inhibitor TGF-β RII Fc. Four dishes of SNs from the same animals were used for each experiment. Each dish was given a different treatment: (i) 5-HT alone, (ii) inhibitor alone, (iii) 5-HT + inhibitor or (iv) Veh alone. Each antagonist was applied to SNs 17 h post-5-HT. The cells were fixed 1 h later for immunofluorescence analysis. For all these experiments, example responses are illustrated and summary data are presented in Fig. 3. Detailed statistics for these experiments are given in Table 1.

Inhibitors were applied just 1 h prior to pERK assay. An observed abrupt decrease in pERK activation might suggest disruption of ongoing positive feedback to maintain elevated pERK.

PKA pathway after the two-pulse protocol**.** 5-HT led to an increase (36.2 ± 10.9%) in levels of pERK (*n* = 6) (Fig. 3A2). This increase was blocked (6.1 ± 6.9%) by RpcAMP (*n* = 6). A one-way RM ANOVA revealed a significant overall effect of the treatments (Table 1). Subsequent pairwise comparisons (Student–Newman–Keuls) analyzed the difference between each pair of four groups (5-HT alone vs. inhibitor alone vs. 5-HT + inhibitor vs. Veh alone). Comparisons revealed that the 5-HT alone group was significantly different from the other three groups. No significant differences were observed between the other three groups.

TrkB pathway after the two-pulse protocol. 5-HT led to an increase (23.9 ± 4.9%) in levels of pERK (*n* = 8) (Fig. 3A3). This increase was blocked (−3.7 ± 4.8%) in the presence of TrkB Fc (*n* = 8). A one-way RM ANOVA revealed a significant overall effect of the treatments (Table 1). Subsequent pairwise comparisons revealed that the 5-HT alone group was significantly different from the other three groups. No significant differences were observed between the other three groups.

**Table 1:** Statistical analysis of pairwise comparisons in Figs 3 and 4

**Fig.** 3**A2 one-way RM ANOVA (F**_**3,15**_ **= 4.273, *P* = 0.023), pairwise comparisons (Student–Newman–Keuls)**			
	*q*	*P*	*n*
5-HT alone vs. Veh group	4.417	0.032	6
5-HT alone vs. RpcAMP alone group	4.209	0.024	6
5-HT alone vs. 5-HT + RpcAMP group	3.604	0.022	6
RpcAMP alone vs. Veh group	0.208	0.885	6
5-HT + RpcAMP vs. Veh group	0.813	0.836	6
RpcAMP alone vs. 5-HT + RpcAMP group	0.605	0.675	6
**Fig.** 3**A3 one-way RM ANOVA (F**_**3,21**_ **= 9.501, p < 0.001), pairwise comparisons (Student–Newman–Keuls)**			
	*q*	*P*	*n*
5-HT alone vs. Veh group	5.986	0.001	8
5-HT alone vs. TrkB Fc alone group	3.746	0.015	8
5-HT alone vs. 5-HT + TrkB Fc group	6.934	<0.001	8
TrkB Fc alone vs. Veh group	2.240	0.128	8
5-HT + TrkB Fc vs. Veh group	0.948	0.51	8
TrkB Fc alone vs. 5-HT + TrkB Fc group	3.187	0.085	8
**Fig.** 3**A4 Friedman repeated measures analysis of variance on ranks (chi-square = 10.714 with 3 degrees of freedom, *P* = 0.013), pairwise comparisons (Student–Newman–Keuls)**			
	*q*	*P*	*n*
5-HT alone vs. Veh group	4.914	<0.05	7
5-HT alone vs. TGF-β RII Fc alone group	3.742	<0.05	7
5-HT alone vs. 5-HT + TGF-β RII Fc group	4.099	<0.05	7
TGF-β RII Fc alone vs. Veh group	3.207	>0.05	7
5-HT + TGF-β RII Fc vs. Veh group	0.535	>0.05	7
TGF-β RII Fc alone vs. 5-HT + TGF-β RII Fc group	2.646	>0.05	7
**Fig.** 3**B2 one-way RM ANOVA (F**_**3,15**_ **= 5.900, *P* = 0.007), pairwise comparisons (Student–Newman–Keuls)**			
	*q*	*P*	*n*
5-HT alone vs. Veh group	4.702	0.012	6
5-HT alone vs. Rp-cAMP alone group	4.591	0.006	6
5-HT alone vs. 5-HT + Rp-cAMP group	5.196	0.011	6
Rp-cAMP alone vs. Veh group	0.112	0.938	6
5-HT + Rp-cAMP vs. Veh group	0.493	0.732	6
Rp-cAMP alone vs. 5-HT + Rp-cAMP group	0.605	0.905	6
**Fig.** 3**B3 one-way RM ANOVA (F**_**3,21**_ **= 8.165, p < 0.001), pairwise comparisons (Student–Newman–Keuls)**			
	*q*	*P*	*n*
5-HT alone vs. Veh group	6.612	< 0.001	8
5-HT alone vs. TrkB Fc alone group	5.287	0.003	8
5-HT alone vs. 5-HT + TrkB Fc group	4.110	0.009	8
TrkB Fc alone vs. Veh group	1.325	0.36	8
5-HT + TrkB Fc vs. Veh group	2.503	0.204	8
TrkB Fc alone vs. 5-HT + TrkB Fc group	1.178	0.415	8
**Fig.** 3**B4 one-way RM ANOVA (F**_**3,21**_ **= 5.950, p = 0.004), pairwise comparisons (Student–Newman–Keuls)**			
	*q*	*P*	*n*
5-HT alone vs. Veh group	4.577	0.011	8
5-HT alone vs. TGF-β RII Fc alone group	3.110	0.039	8
5-HT alone vs. 5-HT + TGF-β RII Fc group	5.601	0.004	8
TGF-β RII Fc alone vs. Veh group	1.467	0.311	8
5-HT + TGF-β RII Fc vs. Veh group	1.023	0.478	8
TGF-β RII Fc alone vs. 5-HT + TGF-β RII Fc group	2.491	0.207	8
Fig. 4B **one-way RM ANOVA (F**_**3,15**_ **= 5.179, *P* = 0.012), pairwise comparisons (Student–Newman–Keuls)**			
	*q*	*P*	*n*
5-HT alone vs. Veh group	3.716	0.047	6
5-HT alone vs. Rp-cAMP alone group	5.401	0.008	6
5-HT alone vs. 5-HT + Rp-cAMP group	3.635	0.021	6
Rp-cAMP alone vs. Veh group	1.685	0.252	6
5-HT + Rp-cAMP vs. Veh group	0.0809	0.955	6
Rp-cAMP alone vs. 5-HT + Rp-cAMP group	1.766	0.444	6
Fig. 4C **Friedman repeated measures analysis of variance on ranks (chi-square = 13.533 with 3 degrees of freedom, *P* = 0.004), pairwise comparisons (Student–Newman–Keuls)**			
	*q*	*P*	*n*
5-HT alone vs. Veh group	4.648	<0.05	9
5-HT alone vs. TrkB Fc alone group	5.333	<0.05	9
5-HT alone vs. 5-HT + TrkB Fc group	3.771	<0.05	9
	*q*	*P*	*n*
TrkB Fc alone vs. Veh group	0.943	>0.05	9
5-HT + TrkB Fc vs. Veh group	3.333	<0.05	9
TrkB Fc alone vs. 5-HT + TrkB Fc group	3.771	<0.05	9
Fig. 4D **Friedman repeated measures analysis of variance on ranks (chi-square = 10.200 with 3 degrees of freedom, *P* = 0.013), pairwise comparisons (Student–Newman–Keuls)**			
	*q*	*P*	*n*
5-HT alone vs. Veh group	5.000	<0.05	9
5-HT alone vs. TGF-β RII Fc alone group	3.873	<0.05	9
5-HT alone vs. 5-HT + TGF-β RII Fc group	5.657	<0.05	9
TGF-β RII Fc alone vs. Veh group	0.000	>0.05	9
5-HT + TGF-β RII Fc vs. Veh group	1.414	>0.05	9
TGF-β RII Fc alone vs. 5-HT + TGF-β RII Fc group	1.000	>0.05	9

TGF-β pathway after the two-pulse protocol**.** 5-HT led to an increase (19.6 ± 3.0%) in levels of pERK (*n* = 7) (Fig. 3A4). This increase was blocked (3.2 ± 4.5%) in the presence of TGF-β RII Fc (*n* = 7). A Friedman repeated measures analysis of variance on ranks revealed a significant overall effect of the treatments (Table 1). Subsequent pairwise comparisons revealed that the 5-HT alone group was significantly different from the other three groups. No significant differences were observed between the other three groups. Thus, inhibitors of PKA, TrkB and TGF-β RII suppressed pERK elevation at 18 h after the two-pulse protocol.

PKA pathway after the standard protocol**.** 5-HT led to an increase (34.1 ± 6.1%) in levels of pERK at 18 h (*n* = 6) (Fig. 3B2). This increase was blocked (2.8 ± 11.6%) in the presence of RpcAMP (*n* = 6). A one-way RM ANOVA revealed a significant overall effect of the treatments (Table 1). Subsequent pairwise comparisons revealed that the 5-HT alone group was significantly different from the other three groups. No significant differences were observed between the other three groups.

TrkB pathway after the standard protocol. 5-HT led to an increase (44.3 ± 13.5%) in levels of pERK at 18 h after the standard protocol (*n* = 8) (Fig. 3B3). This increase was blocked (16.8 ± 5.7%) in the presence of TrkB Fc (*n* = 8). A one-way RM ANOVA revealed a significant overall effect of the treatments (Table 1). Subsequent pairwise comparisons revealed that the 5-HT alone group was significantly different from the other three groups. No significant differences were observed between the other three groups.

TGF-β pathway after the standard protocol. 5-HT led to an increase (21.6 ± 3.6%) in levels of pERK at 18 h after the standard protocol (*n* = 8) (Fig. 3B4). This increase was blocked (−4.7 ± 4.0%) in the presence of TGF-β RII Fc (*n* = 8). A one-way RM ANOVA revealed a significant overall effect of the treatments (Table 1). Subsequent pairwise comparisons revealed that the 5-HT alone group was significantly different from the other three groups. No significant differences were observed between the other three groups. Thus, inhibitors of PKA, TrkB and TGF-β RII suppressed pERK elevation at 18 h after the standard protocol.

### PKA, TrkB and TGF-β pathways mediate the 24-h increase of pERK after the enhanced protocol

We investigated whether the sustained increase of pERK at 24 h after the enhanced protocol is induced by the same pathways as those found to underly the increase of pERK at 18 h after the two-pulse and standard protocols. For these three pathways, each inhibitor was applied to SNs 23-h post-5-HT. The cells were fixed 1 h later for immunofluorescence analysis. Example responses are illustrated and summary data are presented in Fig. 4.

PKA pathway. 5-HT led to an increase (22.3 ± 8.2%) in levels of pERK (*n* = 6). This increase was blocked (0.1 ± 8.3%) in the presence of RpcAMP (*n* = 6). A one-way RM ANOVA revealed a significant overall effect of the treatments (Table 1). Subsequent pairwise comparisons revealed that the 5-HT alone group was significantly different from the other three groups. No significant difference was observed between the other three groups. These results indicate the PKA inhibitor can block pERK elevation at 24 h after the enhanced protocol.

**Figure 5:**
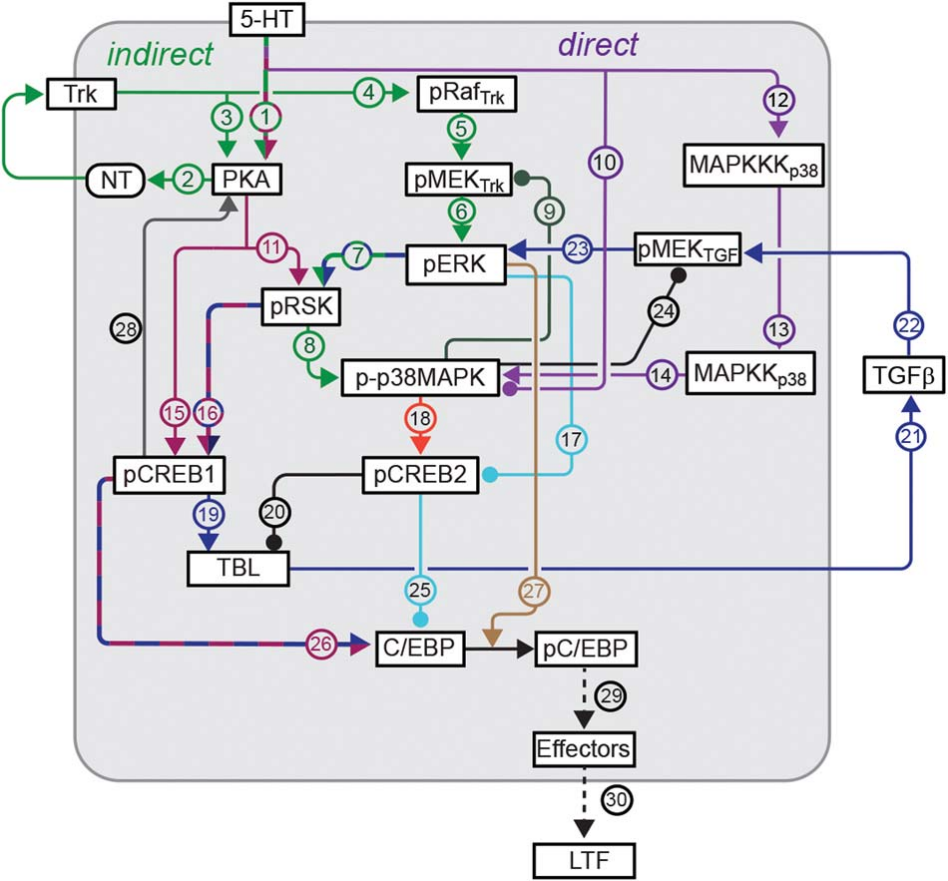
Model for the molecular pathways in SNs that regulate pERK dynamics and LTF. Release of 5-HT activate multiple kinases that are involved in the activation of ERK, including PKA, RSK and p38 MAPK. These kinases interact with growth factors NT and TGF-β to induce multiple feedback loops for the sustained increase of ERK activity up to 24 h after 5-HT. Activation of ERK and other kinases in turn activates genes (e.g. *creb1/2, c/ebp*) essential for the induction of LTF. These processes do not all occur at a single time point, but rather sequentially or, in some cases, partly in parallel. The final steps (dashed lines) between phosphorylation of C/EBP, activation of downstream effector genes and LTF schematically represent numerous, mostly uncharacterized processes. The ultimate output is affected by interlocking positive and negative feedback loops that determine CREB1 and CREB2 activity. Arrowheads indicate activation, circular ends indicate repression.

TrkB pathway. 5-HT led to an increase (43.6 ± 12.4%) in levels of pERK (*n* = 9). This increase was reduced but still significantly increased from the basal (24.6 ± 7.9%) in the presence of TrkB Fc (*n* = 9). Friedman repeated measures analysis of variance on ranks revealed a significant overall effect of the treatments (Chi-square = 13.533 with 3 degrees of freedom, *P* = 0.004, *n* = 9). Subsequent pairwise comparisons revealed that the 5-HT alone group was significantly different from the Veh, TrkB Fc alone and 5-HT + TrkB Fc groups (Table 1). The 5-HT + TrkB Fc group was significantly different from Veh and from TrkB Fc alone. No significant difference was observed between TrkB Fc alone and Veh. These results indicate that TrkB Fc can reduce, but not completely block, pERK elevation at 24 h after the enhanced protocol. This result contrasts with the more complete block of pERK elevation by TrkB Fc found above at 18 h post 5-HT.

TGF-β pathway. 5-HT led to an increase (34.7 ± 15.2%) in levels of pERK (*n* = 9). This increase was blocked (7.8 ± 6.7%) in the presence of TGF-β RII Fc (*n* = 9). A Friedman repeated measures analysis of variance on ranks revealed a significant overall effect of the treatments (Table 1). Subsequent pairwise comparisons revealed that the 5-HT alone group was significantly different from the other three groups. No significant difference was observed among the other three groups. Thus, TGF-β RII Fc suppressed pERK elevation at 24 h after the enhanced protocol.

## DISCUSSION

Immunofluorescence analysis was used to characterize the dynamics of activation of ERK up to 24 h after different 5-HT protocols. The effects of different inhibitors suggest that multiple pathways and feedback loops contribute to the long-term activation of ERK (Fig. 5). Differential pERK dynamics observed during the late consolidation phase provided insights into a mechanism that could contribute to the effectiveness of the two-pulse protocol in producing LTF and also contribute to the effectiveness of the enhanced protocol in producing greater and more persistent LTF than the standard protocol [16].

## PHOSPHORYLATION OF ERK HAS COMPLEX DYNAMICS

Empirical studies identified three different 5-HT protocols that can induce LTF. Two protocols have been identified previously: the standard protocol and the enhanced protocol. The two-pulse protocol was shown in this paper to induce LTF as well (Fig. 1), consistent with a recent finding of Kukushkin *et al.* [51] and with the ability of two sensitization trials to induce LTM [45]. The Standard and two-pulse protocols led to an immediate increase in pERK, which decayed within 5 h post treatment. A second wave of increased pERK was detected at 18 h post treatment. This late phase was blocked by RpcAMP, and by the antagonists TrkB Fc and TGF-β RII Fc. For all three of these inhibitors, this block was assayed just 1 h after inhibitor application 17 h post treatment. These dynamics suggest that late pERK activity, necessary for maintenance of LTM, may be sustained by positive feedback that is susceptible to rapid disruption by inhibitors of the PKA, TrkB or TGF-β signaling pathways, all previously implicated in the formation of LTM. PKA activity is increased 20 h after the standard protocol [22]. The results here suggest that PKA activity might already be elevated at 18 h, to directly or indirectly activate TrkB and TGF-β, leading to the increase of pERK (Fig. 5, pathways 1 (green/red) → 2 (green) → 4 (green) → 5 (green) →  6 (green); and 1 (green/red) → 15 (red) → 19 (blue) → 21 (blue) → 22 (blue) → 23 (blue)). The PKA holoenzyme consists of a tetramer of regulatory (R) and catalytic (C) subunits, and cAMP activates PKA by binding to the R subunits and dissociating them from C subunits. RpcAMP also binds to R and prevents cAMP from binding and C from dissociating [52]. Therefore, the observed effectiveness of RpcAMP in inhibiting a PKA effect at 17 h post-5-HT implies that R subunits must have been present at that time. Thus, although regulatory subunit levels do decrease after 5-HT application [53], at 17 h a substantial level of regulatory subunits must still remain or have been re-synthesized after degradation. It is likely that rich dynamics of pERK will be shared by other kinases and transcription factors given the complexity of the metabolic circuit (Fig. 5). A recent study has further characterized the dynamics of phosphorylation of specific sites on *Aplysia* ERK induced by two 5-HT pulses 45 min apart, using antibodies with differential site specificity [51].

To further clarify whether persistent activation of these signaling pathways is necessary for maintaining LTM, it will be important in future studies to test whether LTF assayed at times including 18 or 24 h, or as late as 5 d, post-treatment is disrupted by late (e.g. 17 h post treatment) inhibition of ERK, as well as by inhibition of the PKA, TrkB, or TGF-β cascades. Given that multiple investigations have established that ERK activation and nuclear translocation are necessary for LTF [9, 20, 23], and that phosphorylation of C/EBP by ERK inhibits the degradation of C/EBP, prolonging its ability to support LTF/LTM [54], we predict that inhibiting any of these pathways at late times will block maintenance of LTF. Blocking these pathways at 18 h is predicted to reduce the strength of LTF induced by the two-pulse protocol at 2 d post-treatment, whereas blocking these pathways at 24 h is predicted to reduce the strength of LTF induced by the enhanced protocol at 5 d post-treatment, although these predictions need to be tested.

## MULTIPLE FEEDBACK LOOPS OF THE ERK, PKA, TRKB AND TGF-Β CASCADES CONTRIBUTE TO THE DYNAMICS OF LONG-TERM ERK ACTIVATION.

Müller and Carew [22] observed three phases of PKA activation after 5-HT – early, intermediate, and late (lasting ~5 min, ~3 h and up to ~20 h, respectively). The intermediate phase is likely due to the PKA – NT – TrkB – PKA positive feedback loop (Fig. 5, green pathways 2 → 3) [19]. The late phase is likely induced by a CREB – *Aplysia* ubiquitin carboxyl-terminal hydrolase (Ap-Uch) – PKA feedback loop [55, 56]. The present study discovered a complex dynamic pattern of ERK activation. Two waves of increase in pERK were induced by the two-pulse and standard protocols. The first wave returned to the basal level within 5 h after 5-HT, whereas the second wave occurred around 18 h after 5-HT. These dynamics were similar to those of PKA activation after the standard protocol [22]. Application of inhibitors suggests that the late increases of pERK induced by the standard and two-pulse protocols are dependent on the PKA, TrkB and TGF-β pathways. We posit that the late phase of PKA activation directly activates the PKA – NT – TrkB – PKA positive feedback loop (Fig. 5, green pathways 2 → 3) and indirectly activates the TGF-β – ERK feedback loop via phosphorylation of CREB1 (Fig. 5, pathways 15 (red) → 19 (blue) → 21 (blue) → 22 (blue)). Both feedback loops contribute to the late increase of pERK at 18 h (Fig. 5, green pathways 4 → 5 → 6; blue pathways 22 → 23). Sustained increase of ERK and PKA pathways converge to regulate CREB1 and CREB2 (pathways 15 (red), 7 (green/blue) → 16 (red/blue), 7 (green/blue) → 8 (green) → 18 (orange), 17 (light blue)) [24, 25], which subsequently increase the expression of C/EBP, essential for the induction of LTF (pathways 25 (light blue), 26 (red/blue)) [57–59]. The late phase of PKA activation relies, as illustrated, on a CREB1-dependent pathway (Fig. 5, pathway 28, CREB1 → PKA). In this pathway (details not shown), pCREB1 activates transcription of Ap-Uch, which enhances degradation of the regulatory subunit of PKA, thereby increasing levels of free, constitutively active catalytic subunit [55, 56].

## PUTATIVE MECHANISM UNDERLYING THE SUPERIORITY OF THE ENHANCED PROTOCOL IN PRODUCING PROLONGED LTF AND IMPLICATIONS FOR TWO-BLOCK TRAINING PROTOCOLS

The enhanced protocol produces LTF that is greater and longer lasting than the standard protocol [16]. Specifically, at 24 h post training, LTF induced by the enhanced protocol is greater than that induced by the standard protocol. Moreover, LTF induced by the standard protocol only persists for ~24 h, whereas LTF induced by the enhanced protocol lasts up to 5 d. The standard protocol in the presence of inhibitors of RSK fails to induce LTF, whereas the enhanced protocol still can induce LTF after the RSK inhibitor is applied [28]. The superiority might be, at least partially, related to the finding that pERK remained elevated at 24 h only after the enhanced protocol. This may reflect an enhanced engagement of the CREB- TGF-β – ERK feedback loop, extending ERK activation and downstream expression of C/EBP (Fig. 5, pathway 26 (red/blue)) and activity of C/EBP (Fig. 5, brown pathway 27), which will lead to an enhanced and prolonged LTF (Fig. 5, dashed black pathways 29➜30). This 24-h ERK increase is abrogated by inhibitors of TGF-β or PKA applied at 23 h, but only partly blocked by inhibition of TrkB (Fig. 4).

LTF has been measured at 48 h, 72 h, and even 7 d after training [16, 60–63]. Two or more blocks of training, or of 5-HT treatment, have been found to prolong LTM and LTF. Each block consists of a standard protocol, and the interval between blocks is ~24 h. The present results predict that use of the enhanced protocol for the first block, or both blocks would produce even stronger LTM, because the second block would overlap with the 24 h ERK increase induced by that protocol.

## SUMMARY AND PERSPECTIVE

The results of the study will provide valuable data for the development of more advanced computational models of the molecular cascades underlying long-term plasticity by us and others. Data from the present study will provide biological constraints to the models, which will, in turn, provide predictions (e.g. an optimal time to apply a second block of 5-HT) that can be empirically tested. Although the present studies focus on LTF, complex dynamics are a ubiquitous feature of molecular cascades (e.g. Smolen *et al.* [64]). For example, in both *Aplysia* and mammals, the activation of PKA and ERK is required for the induction of LTM. This study further suggests that prolonged ERK activation, concurrent with trial-induced PKA activity, is important for the maintenance of some forms of LTM, at least over an ~1 d timescale. We note that recent studies have determined that persistent activation of additional kinases, the truncated protein kinase C isoforms denoted protein kinase M Apl I–III, is also necessary to maintain LTF at later times (> 3 d post-stimulus) [65, 66]. The role of active ERK at these later times remains to be determined.

In rodents, intra-hippocampal infusion of PKA inhibitor immediately after training results in amnesia, whereas infusion of an ERK inhibitor does not. The ERK inhibitor is effective 3 h post-training, however [67, 68]. Molecular processes such as the activity of protein phosphatase 1 decode the temporal features of stimulus protocols that induce long-term potentiation (LTP) or long-term depression (LTD) [69]. Spaced training enhances ERK activation and LTP [70, 71]. In *Drosophila*, the relative timing of activation of MAPK and of phosphatase by spaced learning protocols, with resultant generation of waves of MAPK activity by successful spaced protocols, plays a key role in determining learning efficacy [72]. Because of the conservation of the molecular processes involved in LTF and late LTP between *Aplysia* and mammals, the findings of this study highlight the importance of examining the dynamics of kinase cascades in neurons involved in long-term memory in vertebrates. A knowledge of these dynamics may provide insights into mechanisms underlying late LTP and help design protocols to enhance memory in mammals.

## FUNDING

This study was supported by U.S.A. National Institutes of Health (NIH) grant NS019895.

## CONFLICT OF INTEREST

None declared.

## Supplementary Material

Web_Material_kvac014
