# Supplementary material for: Dynamics and Mechanisms of ERK Activation after Different Protocols that Induce Long-Term Synaptic Facilitation in Aplysia
Source: Oxf Open Neurosci. 2022 Oct 18;2:kvac014. doi: 10.1093/oons/kvac014 (PMC10464504; doi:10.1093/oons/kvac014)
Supplement: Web_Material_kvac014 [file Web_Material_kvac014.zip › Zhang_et_al_2022_Oxford_response letter_v2.docx]

Department of Neurobiology and Anatomy

John H. Byrne, Ph.D.

*Professor and June and Virgil Waggoner Chair*

October 5th, 2022

Carlos Ibanez, PhD

Professor, Peking University & Karolinska Institute

Senior Editor, *Oxford Open Neuroscience*

Dear Dr. Ibanez,

Attached is our revised manuscript entitled “Dynamics and mechanisms of ERK activation after different protocols that induce long-term synaptic facilitation in *Aplysia*” (manuscript number OXFNSC-2022-013). The two Reviewers provided helpful comments, and the manuscript was revised accordingly. A point-by-point reply to the Reviewers' comments is provided below. Page and figure numbers refer to the revised manuscript, unless otherwise indicated. Significant text revisions and newly added references are in red font in the revised manuscript.

**Reviewer #1**

Major Comment:

1. Although not marked as a concern, the reviewer makes a substantial suggestion for improvement as follows, “*..perhaps the most informative result in the manuscript is the fact that pERK at 18 h can be returned to baseline by applying the inhibitors only an hour before fixation, i.e. 17 hours after 5HT treatments. This strongly suggests a cyclical, self-sustaining loop of cellular activation that persists long after the initial stimuli and has the capacity to re-engage the very same mechanisms that set it in motion. (There are parallels between this study and Miyashita, Cell Rep 2018, which might be worth mentioning.) Although this result is clear from careful reading, I would suggest that the authors emphasize it in stronger terms, specifically referring to the timing of inhibitor treatments”*

Response: We now emphasize the putative role of positive feedback in sustaining ERK activation, and the supporting evidence that inhibitor treatments at 17 h abruptly returns pERK to baseline at 18 h. See highlighted text on pp. 2, 3, 13, and 17.

Minor Comments:

1. *As mentioned above, a stronger emphasis on the abrupt effect of inhibitors, applied following a prolonged rest period, would help advance the argument of the paper.*

Response: Dealt with as response to Major Comment above.

2. *In general, the manuscript could benefit from being condensed. At the moment, each protocol and each inhibitor treatment is reported in a separate manuscript section, making it difficult to perceive the results as a whole (the figures, however, are illustrative and easy to understand). Some possible ways to “unload” the text could be reporting summary statistics in a separate supplementary table, and/or discussing each experiment with reference to all three protocols at the same time, in standardized order that would be easy to follow.*

Response: Substantial work has been done to condense the experimental sections on pp. 13-16. The summary statistics are now reported in a separate table. Text describing inhibitor applications, which was previously repeated for each experiment, has been combined into the new highlighted statements on pages 13 and 15.

3. *The obvious next step for this study would be to investigate whether the same manipulations that block pERK induction 18/24 h after 5HT treatments would also block LTF at the 18–24 h (or perhaps later) time points. It might be a good idea to articulate the predictions for such experiments. (At present, the one stated prediction is that blocking pERK 22–24 h after training should render the Enhanced protocol equivalent to the Standard protocol when tested at later time points. What about two-trial LTF at 24 h when pERK is blocked at 18 h?)*

Response: On pp. 18-19 we have added a paragraph to address this suggestion. The paragraph begins, “To further clarify whether persistent activation of these signaling pathways is necessary for maintaining LTM, it will be important in future studies to test whether LTF assayed at times including 18 or 24 h, or 5 d, post-treatment is disrupted by late (e.g. 17 h post treatment) inhibition of ERK, as well as by inhibition of the PKA, TrkB, or TGF-β cascades.” New predictions are added in this paragraph.

4. *The “Lay summary” largely restates the abstract and might be too complex for a lay reader. In my opinion, the paper is sufficiently deep to provide an outside reader with a “big picture”.*

Response: We have completely rewritten the Lay Summary to be more engaging and accessible to a general audience of biologists. The putative role of positive feedback is now emphasized.

5. *MAPK is a family of proteins rather than a particular protein type; thus ERK is not generally considered a “MAPK isoform” (for example, mammalian ERK1 and ERK2 are isoforms, but ERK1 and p38 are distinct proteins).*

Response: “MAPK isoform” has been changed to “MAPK family member” throughout.

**Reviewer #2**

Major Comments:

1. *Conceptual context. The presentation of the rationale for these experiments and the description of these results focus largely on details. For example, the Results point out that “there is a lack of comparison of detailed dynamics of long-term activation of ERK by different protocols and how ERK activity is regulated by growth factors and kinases that interact with MAPK pathways.” And the Discussion summarizes the objective as characterizing “the dynamics of activation of ERK up to 24 h after different 5-HT protocols.” Thus the emphasis is on ERK dynamics. However, this misses an opportunity to emphasize novel aspects of these findings. By the mid-1980s, it was believed that in Aplysia, protein synthesis was important for initiating long-term facilitation within a few hours after training or 5-HT exposure, but not at 24 hours for maintenance of plasticity (based on Montarolo et al., 1986). Subsequently, Chain et al (1999) reported that persistent PKA activity was important several hours after training, but not after 12 hours. A simplified view of the induction and maintenance of long-term synaptic plasticity during memory formation is that initially, protein kinases play essential roles, followed by transcription cascades and protein synthesis. C/EBP is considered an immediate early gene. (Of course, in Aplysia sensory neurons, protein synthesis is required for the persistent activation of PKA resulting from degradation of the regulatory subunit of PKA.) In this context, the results describing ongoing increases in MAPK activation at 24 hr, and the importance of PKA activity in driving this activation at both 18 and 24 hr, are somewhat surprising. The Abstract and Discussion ignore these novel aspects and the role of persistently active kinases. PKM, and its role in the maintenance of memory in both Aplysia and mammals, are not mentioned.*

Response: For the Abstract, we have added text briefly describing the novelty of long-lasting ERK activation and its role in LTF, and plausible role of positive feedback, “These results begin to characterize novel, long-lasting ERK activation, plausibly maintained in part by positive feedback involving growth factors and PKA.”

For the Discussion, we have added text on p. 21 describing the role of PKM isoforms in maintaining late memory in *Aplysia*. As the role of PKM in mammals remains controversial, we have avoided a statement here. New text has also been added on pp. 18-19, predicting that late activation of ERK, PKA, and the TrkB and TGF-β pathways are likely to be important for maintaining LTF, but emphasizing that this prediction will need to be tested.

2. *The Lay Summary fails to emphasize general principles that would be widely understood. Rather it provides details of the activation of signaling cascades, with language excerpted from the Abstract. The Lay Summary should be accessible for those who know nothing of these various signaling cascades (probably focusing on signaling feedback loops and protein synthesis). The Lay Summary does not mention the 24 hr time point w selective elevation pf MAPK activity enhanced.*

Response: We have completely rewritten the Lay Summary to be accessible to a general audience of biologists, and positive feedback is now emphasized. The 24 h time point of ERK activation is noted. Due to length constraints (the new Summary is 1 word short of the allowed maximum) and the new focus on accessibility, differences between the three 5-HT protocols are not described.

Minor Comments:

1. *The manuscript focuses on the persistence of MAPK activation when the real question is persistence of memory. Are signaling cascades required to remain active at 25 hr for LTF to persist? This question should be discussed. Additional experiments are not required for this paper, but it would be worth proposing a follow up experiment – such as testing the effect of late MAPK inhibition on LTF at 18 or 24 hours.*

Response: On pp. 18-19 we have added a paragraph to address this concern. The paragraph begins, “To further clarify whether persistent activation of these signaling pathways is necessary for maintaining LTM, it will be important in future studies to test whether LTF assayed at times including 18 or 24 h, or 5 d, post-treatment is disrupted by late (e.g. 17 h post treatment) inhibition of ERK, as well as by inhibition of the PKA, TrkB, or TGF-β cascades.”

2. *What does pERK signify? The point that ERK phosphorylation is a substitute for measuring ERK activity is not clearly explained.*

Response: We now define pERK at the last line of p. 4 – top of p. 5 as follows, “…ERK that has been activated by obligatory phosphorylation, which we denote pERK “

3. *Both the Abstract, the Lay Summary and the Introduction state that the “late phase was blocked by inhibitors of protein kinase A, TrkB and TGF-beta.” However, depending on the section, it seems that the late phase may be the 18 hr time point or the 24 hr time point, or both. This should be clarified.*

Response: In the Abstract, the results with the 24 h time point are now clearly distinguished from those with the 18 h time point. In the Introduction (p. 5) we also now clearly distinguish the 18 h and 24 h results.

4. *Methods – There is information provided about the volume of bath washes after 5‑HT exposure, but no information about the dish volume.*

Response: The dish volume is now given on p. 7.

5. *What was fixation time for immunofluorescence?*

Response: The fixation times are now given on p. 7.

6. *Results – The introduction to the two pulse protocol explains that the first 5-HT pulse recruits nuclear MAPK activity, CREB1 kinase and C/EBP. What is “CREB1 kinase” (this is mentioned only a single time in the MS)? What signaling processes are recruited by the second 5-HT pulse?*

Response: On p. 10, we now specify that p90 ribosomal S6 kinase is the CREB1 kinase, and we now briefly discuss that a second 5-HT pulse at 45 min recruits PKA at a time when MAPK activity is still elevated, facilitating overlap of MAPK and PKA activities that are thought to be important for LTF. A downstream effector of PKA that is needed for LTF is also noted.

7. *PKI vs. Rp-cAMPS (assuming this is the substituted inhibitor). Chain et al. used the peptide inhibitor of PKA, which is effective after the decrease in regulatory subunits during LTF. It is somewhat surprising that the application of Rp-cAMPS at 17 hours after 5-HT exposure eliminates the persistent activation of ERK. Would an inhibitor of the regulatory subunit be expected to be this effective after the regulatory subunits have been degraded? (However, see Greenberg et al. 1987.)*

Response: We have added text on p. 18 to address this concern to the extent possible: “…Therefore, our observed effectiveness of RpcAMP in inhibiting a PKA effect at 17 h post – 5-HT implies that R subunits must have been present at that time. Thus, although regulatory subunit levels do decrease after 5-HT application (Greenberg et al. 1987), at 17 h a substantial level of regulatory subunits must still remain, or have been re-synthesized after degradation. “

8. *It seems that TrkB is only partially responsible for the increase in ERK activity 24 hr after 5-HT exposure, but ERK activation is more completely dependent on TrkB signaling 18 hr after 5-HT. It is not clear that this distinction was communicated in the Discussion or Abstract.*

Response: We now state in the legend to Fig. 4, and on p. 20 in the Discussion, that the dependence of ERK activation on TrkB at 24 h is partial. We have also added a sentence on p. 16, “This result contrasts with the more complete block of pERK elevation by TrkB Fc found above at 18 h post 5‑HT.” However, we note that the 24 h result is with the Enhanced 5-HT protocol whereas the 18 h results are with the Standard and two-pulse protocols, preventing a direct comparison.

9. *There are two statements (in Results and Discussion) about LTF with the two-pulse protocol. “The two-pulse treatment lead to a significant enhancement of the EPSPs.” (perhaps this should be led?). “The two-pulse protocol was shown in this paper to induce LTF as well.” This appears to be a novel finding. However, it is consistent with the long-term sensitization finding of Philips et al. (2007) using the two-trial protocol; the findings should be compared.*

Response: The grammatical error has been corrected in the first statement. On p. 17, we now state that the induction of LTF by two pulses is consistent with the long-term sensitization observed by Philips et al. (2007) with two trials. We also mention a new paper by Kukushkin et al. 2022 showing LTF induction using a two-pulse protocol.

10. *The sentence “could contribute the effectiveness of the Enhanced protocol in producing greater and more persistent LTF than the Standard protocol” is missing “to,” and also doesn’t mention the two-pulse protocol.*

Response: We have added the two-pulse protocol to this sentence (p. 17) and corrected the grammatical error.

11. *The statement in the Discussion, “in both Aplysia and mammals, the activation of PKA and ERK is required for the induction of LTM” ignores what is unusual about the current findings, that PKA and ERK activity may be required for maintenance of memory, at least at 18 or 24 hr.*

Response: A new paragraph has been added to the Discussion on pp. 18-19, in part to address this point. The paragraph begins by stating, “To further clarify whether persistent activation of these signaling pathways is necessary for maintaining LTM, it will be important in future studies to test whether LTF assayed at times including 18 or 24 h, or 5 d, post-treatment is disrupted by late (e.g. 17 h post treatment) inhibition of ERK, as well as by inhibition of the PKA, TrkB, or TGF-β cascades.“

12. *Fig. 2A. The legend and the Results should state whether the difference between pERK at 18 hr is significantly different between the two pulse protocol and the standard and enhanced protocols.*

Response: We now state in the Results (p. 12) and in the legend to Fig. 2 that there were no significant differences between pERK levels at 18 h induced by the two-pulse, Standard, and Enhanced protocols.

13. *In Fig. 2, because panel A has multiple time points, it would be helpful to include the label 24 h on the panel or panels in B.*

Response: The 24 h label has been added to Fig. 2B1 as requested.

14. *The legends for Figs. 3 and 4 refer to “nonoutliers.” This should be clearly explained.*

Response: The legends for Figs. 3 and 4 were revised to clarify that the ends of the vertical lines (whiskers) in Figs. 3A4, 4C-D are the maximum and minimum values of all data points. We verified there are in fact no outliers in these data.

15. *Fig. 4 legend title “Late increase of pERK at 24 h after the Enhanced protocol was dependent on PKA and TGF-beta.” This should probably include “and partially dependent on TrkB.”*

Response: The suggested phrase has been added to the legend of Fig. 4.

16. *In Fig. 5, the steps between C/EBP and Effectors and Long-term facilitation are totally unknown. Somehow, this should be emphasized graphically and in the legend. There may be other points in the model for which there is less empirical evidence; these should be distinguished by color or line type. Some aspects of this diagram are oversimplified, as they do not occur synchronously, at a single time point. This point should be made in the legend and possibly the Discussion.*

Response: The Fig. 5 legend has been expanded to a) state that numerous uncharacterized processes occur between C/EBP, Effectors, and LTF, and b) state that processes in the model do not all occur at a single time point, but rather sequentially or in some cases in parallel. Differences in the strength of empirical evidence for the pathways shown are graded and to some extent subjective, so that we have not found a useful way to illustrate these graphically.

17. *The model in Fig. 5 would benefit from inclusion of the degradation of the regulatory unit of PKA. This is key to the persistence of PKA activation.*

Response: We have added a simplified representation of 5-HT – induced, CREB1 – dependent degradation of PKA to Fig. 5. In the manuscript text, we now give some details of this pathway and references on p. 20.

The comments of the Reviewers were extremely helpful, and we believe the manuscript has been improved by addressing the Reviewers' concerns. We hope that you find the revised manuscript suitable for publication, and we thank you for your consideration.

Thank you for your consideration.

Sincerely,

John H. Byrne
